# Topical Loperamide-Encapsulated Liposomal Gel Increases the Severity of Inflammation and Accelerates Disease Progression in the Adjuvant-Induced Model of Experimental Rheumatoid Arthritis

**DOI:** 10.3389/fphar.2017.00503

**Published:** 2017-08-02

**Authors:** Susan Hua, Thilani H. Dias, Debbie-Gai Pepperall, Yuan Yang

**Affiliations:** ^1^School of Biomedical Sciences and Pharmacy, University of Newcastle Callaghan, NSW, Australia; ^2^Hunter Medical Research Institute New Lambton Heights, NSW, Australia; ^3^Centre for Inflammatory Diseases, Monash University Melbourne, VIC, Australia

**Keywords:** loperamide, arthritis, opioid, peripheral opioid, inflammation, pain, liposomes

## Abstract

This study evaluates the prophylactic effect of the peripherally-selective mu-opioid receptor agonist, loperamide, administered topically in a liposomal gel formulation on pain, inflammation, and disease progression in the adjuvant-induced model of experimental rheumatoid arthritis in female Lewis rats. In a randomized, blinded and controlled animal trial, AIA rats were divided into six groups consisting of eleven rats per group based on the following treatments: loperamide liposomal gel, free loperamide gel, empty liposomal gel, diclofenac gel (Voltaren®), no treatment, and naive control. Topical formulations were applied daily for a maximum of 17 days—starting from day 0 at the same time as immunization. The time course of the effect of the treatments on antinocieption and inflammation was assessed using a paw pressure analgesiometer and plethysmometer, respectively. Arthritis progression was scored daily using an established scoring protocol. At the end of the study, hind paws were processed for histological analysis. Administration of loperamide liposomal gel daily across the duration of the study produced significant peripheral antinociception as expected; however, increased the severity of inflammation and accelerated arthritis progression. This was indicated by an increase in paw volume, behavioral and observational scoring, and histological analysis compared to the control groups. In particular, histology results showed an increase in pannus formation and synovial inflammation, as well as an upregulation of markers of inflammation and angiogenesis. These findings may have implications for the use of loperamide and other opioids in arthritis and potentially other chronic inflammatory diseases.

## Introduction

Rheumatoid arthritis (RA) is an incurable, systemic autoimmune disease that is primarily manifested by chronic erosive inflammation of the joints, associated with pain. Despite major advances in treatment strategies over the last two decades, pain management still remains a major challenge in arthritis (Heiberg and Kvien, 2002; Minnock et al., 2003). Many patients with RA continue to experience severe pain even when inflammation is well-controlled (Kvien, 2004; Stein and Baerwald, 2014; Wigerblad et al., 2016). Since the development of the three-step pain relief ladder by the World Health Organization, there has been widespread use of opioids in chronic non-cancer pain (CNCP)—including in RA patients (Stein and Baerwald, 2014). Only a few randomized, controlled clinical studies dealing with opioids in RA have been reported. These were only focused on analgesic outcomes and concluded that opioids were superior to placebo, but were hampered by severe side effects (Whittle et al., 2011, 2013). Despite this, the percentage of RA patients who are prescribed opioids is still increasing (Grijalva et al., 2008; Katz, 2008).

Systemically administered opioids (e.g., morphine) act on both central and peripheral opioid receptors to elicit pain relief (Hua and Cabot, 2010; Iwaszkiewicz et al., 2013). The effects of opioids on central opioid receptors are well-described, and are associated with the well-known side effects of opioids (e.g., sedation, tolerance, and dependence). As agonists acting on peripheral opioid receptors do not elicit these central effects, current research has focused on administering opioids locally (e.g., topical and intra-articular injection) to achieve analgesia (Stein et al., 2003; Hua and Cabot, 2010; Iwaszkiewicz et al., 2013). A number of preclinical and clinical studies have demonstrated that opioids display potent peripheral analgesic effects in various types of pain (Stein et al., 2003; Hua and Cabot, 2010; Iwaszkiewicz et al., 2013). However, the direct effects of opioids on peripheral tissues and cells, especially in chronic inflammatory diseases such as RA, are not fully understood. In particular, the effect of peripheral opioids on inflammation has only recently been studied, with results showing potentially a variety of complex regulatory activities in various tissues of the body (Philippe et al., 2003; Smith et al., 2007; Stein and Kuchler, 2012; Iwaszkiewicz et al., 2013). No clinical data is currently available on the peripheral effect of opioid use in patients with chronic inflammatory disorders, in particular RA, due to a lack of clinical studies designed to focus on this outcome.

In this study, we evaluated the prophylactic effect of the peripherally-selective mu-opioid receptor (MOR) agonist, loperamide, administered topically in a liposomal gel formulation on pain, inflammation, and disease progression in the adjuvant-induced model of experimental RA. Loperamide is clinically used as an antidiarrhoeal agent due to its peripheral selectivity. Although it has strong affinity and high selectivity for MORs, it does not have analgesic effects when administered orally, intravenously, or topically (on intact skin) due to its physicochemical properties (Alyautdin et al., 1997; DeHaven-Hudkins et al., 1999; Hagiwara et al., 2003; Menendez et al., 2005; Sevostianova et al., 2005; Hua and Cabot, 2010). In order to allow the study of loperamide as an analgesic agent, we have developed liposomal delivery systems to bypass biological barriers and allow the release of loperamide at sites of tissue injury. Our work has previously demonstrated both antinociceptive and anti-inflammatory actions following intravenous (Hua and Cabot, 2013) and topical (Iwaszkiewicz and Hua, 2014) administration of liposomal loperamide in the Complete Freund's Adjuvant (CFA) rodent model of acute inflammatory pain. Hence the hypothesis of this study is that chronic topical application of our liposomal loperamide formulation would lead to similar antinociceptive and anti-inflammatory effects in a chronic inflammatory pain model—in this case a rodent model of experimental RA.

## Materials and methods

### Preparation of liposomes

Liposomes were prepared according to the method of dried lipid film hydration. Briefly, 96 mg L-α-phosphatidylcholine (EPC) (Avanti Polar Lipid, Alabama, USA) and 24 mg cholesterol (molar ratio of 2:1) (and 24 mg loperamide HCl) (Sigma-Aldrich, Sydney, Australia) were solubilized in 6 ml chloroform:methanol (2:1, v/v) in a 50 ml round bottomed flask and dried by rotary evaporation under reduced pressure (100 mbar; 10 min; 37°C). In addition, 60 μl of 1,1′-dioctadecyl-3,3,3′,3′-tetramethylindocarbocyanine perchlorate (DiI) (Invitrogen, Victoria, Australia) was added to tag the liposomes pink so that an even dispersion in the gel could be visually gauged. The resultant thin lipid film was hydrated with the addition of 6 ml of sterile phosphate buffered saline (PBS; pH 6.5) and resuspended in a 37°C water bath. The resultant multilamellar liposomes were then reduced in lamellarity and size to 100 nm via probe sonification (60 amps, 10 mins, 37°C). The size distribution of the liposomal dispersion was determined by dynamic laser light scattering (Zetasizer Nano S™, ATA Scientific). Unencapsulated drug was removed from the liposome suspension using Slide-A-Lyser dialysis cassettes with a 10 kDa MWCO (Thermo Fisher Scientific, Scoresby, Victoria) at 4°C. Encapsulation efficiency (EE%) was determined by disrupting the vesicles with ethanol and evaluating loperamide HCl concentration using HPLC. Loperamide-encapsulated liposomes had a mean particle size of 102 nm and a polydispersity index of 0.203. The size and polydispersity of the control liposome formulation was similar. A low PDI (<0.3) signifies that the mean particle size is an adequate indicator of the size variance in the entire sample. This procedure resulted in high loperamide HCl encapsulation efficiency of >99%, which equated to 3.86 ± 0.068 mg (mean ± SD) of loperamide HCl encapsulated in each milliliter of the liposome suspension. Drug release assays for the loperamide-encapsulated liposomes have previously been conducted with consistent results (Hua, 2014; Iwaszkiewicz and Hua, 2014). Liposomes were stored at 4°C and were used within 14 days. Our laboratory has previously confirmed that the liposomes are stable in size, polydispersity, and loperamide concentration over this time period (Iwaszkiewicz and Hua, 2014). All chemicals and solvents were of at least analytical grade.

### Preparation of loperamide-encapsulated liposomal gel

Carbopol® gel was prepared by dispersing 1% (w/w) carbomer 940 NF resin (PCCA, Houston, USA) in sterile distilled water (44 g), in which glycerol (5 g) was previously added. The mixture was stirred until thickening occurred and then neutralized by the drop wise addition of 50% (w/w) triethanolamine to achieve a transparent gel of pH 5.5. Prior to addition of the liposomes to the Carbopol® gel, superfluous liquid was removed from the liposome suspension to prevent decreased viscosity of the gel. The initial volume of 6 ml was spun down in an ultrafiltration centrifuge tube (Thermo Fisher Scientific, Scoresby, Australia) at 2,500 rpm for 1 h to achieve a final volume of 760 μl. Encapsulation efficiency was again analyzed via HPLC. The liposome suspension was then added to 2.24 ml 1% (w/w) Carbopol® gel which equated to a loperamide HCl concentration of ~8 mg/mL. Liposomes were mixed into the Carbopol® gel by manual stirring for 5 min to ensure a homogenous dispersion. Empty liposomes were made following the same method, without the addition of loperamide HCl. Free loperamide gel (free drug mixed into a gel base) was manufactured by the addition of 16 mg of loperamide HCl to 2 ml of 1% (w/w) Carbopol® gel, in order to keep the concentration of loperamide consistent with the liposomal formulation. The diclofenac gel was purchased commercially [Voltaren®, 1% (w/w) diclofenac sodium].

### HPLC analysis of loperamide HCl

The concentration of loperamide HCl was evaluated via HPLC (Agilent Technologies 1200 series HPLC system). Separation was performed using a Thermo Scientific BDS Hypersil C18 column (150 × 4.6 mm, 5 μm), which was maintained at a temperature of 25°C and with a detection wavelength of 210 nm. The mobile phase was pumped through the column at a flow rate of 1.5 ml/min and consisted of 5% isopropanol, 50% acetonitrile, and 45% buffer (0.05M NaH_2_PO_4_ pH 4.5). Data was integrated using Agilent Chemstation software. All chemicals and solvents were of at least analytical grade.

### Adjuvant-induced arthritis (AIA) model

Female Lewis rats (6–8 weeks; ARC, Perth, Australia) were used in this study based on established protocols (Whiteley and Dalrymple, 2001), as they are the most susceptible strain to the induction of arthritis with heat-killed *Mycobacterium tuberculosis*. In addition, female rats tend to develop arthritis more readily than male rats, which is similar to what occurs in human rheumatoid arthritis. Animals were housed in standard laboratory cages under control conditions (12-h light-dark cycles, 22°C, 60% humidity) in groups of 4–6, with free access to food and water on tissue and shredded paper bedding. Rats were given a minimum of 7 days to acclimatize to the housing conditions. After this period rats were anesthetized via brief exposure to 2% isoflurane (Abbot, Cronulla, Australia) before receiving a single subcutaneous injection of Complete Freund's Adjuvant (CFA), containing 1 mg heat-killed *M. tuberculosis* (20 mg/ml) (Chondrex, Washington, USA) into the base of the tail. The experiments were approved by The University of Newcastle Animal Care and Ethics Committee.

### *In vivo* study blinding and bias

The *in vivo* studies were conducted as randomized, blinded, and controlled animal trials. Animals were allocated to different treatment groups at random using envelopes containing specifics codes, in order to minimize bias and control variation. The investigator administering the treatments and conducting the preclinical testing throughout the study was blinded to the treatment allocation. A major limitation to the blinding, which we had expected, was that diclofenac 1% gel (Voltaren®) had to be applied three times a day in accordance with the manufacturer's instructions, whereas the other treatments were applied once daily. The main purpose of the study was to evaluate the effect of loperamide liposomal gel in an animal model of chronic inflammatory pain following once daily dosing. This is to compare whether our once daily formulation would be more effective than standard topical NSAIDs, which must be applied three or four times daily for efficacy. The effect of NSAIDs on pain and inflammation is already well-established in the literature and clinic, and we have used it as a positive control in our previous studies (Hua and Cabot, 2013; Iwaszkiewicz and Hua, 2014). In addition, the no treatment and naive control groups also affected the blinding, as they did not receive any treatments throughout the study. Despite these limitations, the most important controls that required blinding in this study were the empty liposomal gel and free loperamide gel groups, in order to assess the effect of the individual components of the loperamide liposomal gel formulation. The loperamide liposomal gel, empty liposomal gel, and free loperamide gel were identical in dosing regimen and appearance. Results of the preclinical testing were also supported by histological analysis to further reduce any bias, and this was performed by investigators blinded to the treatment allocation and hypothesis of the study. The blinded investigators conducting the histological analysis were different to the investigator conducting the preclinical testing.

### *In vivo* study design

A sample size of 11 rats per experimental group was chosen based on previous studies using the established AIA protocol (Binder and Walker, 1998; Whiteley and Dalrymple, 2001; Straub et al., 2008). This allowed statistical analysis with 90% power and a significance level of 0.025 based on a minimal detectible difference of 1.5 standard deviations. Baseline measurements were taken prior to CFA injection and prior to administration of the topical formulations. AIA rats were divided into six experimental groups consisting of 11 rats in each group based on treatment: (i) loperamide liposomal gel, (ii) free loperamide gel, (iii) empty liposomal gel, (iv) diclofenac gel (Voltaren®), (v) no treatment, and (vi) naive control. The control groups were selected for consistency with our previous study in the CFA model of acute inflammatory pain (Iwaszkiewicz and Hua, 2014). Topical formulations were applied before the onset of clinical disease (prophylaxis)—starting from day 0 at the same time as immunization. This set-up was chosen over a therapeutic design (starting the treatment at day 9) as we wanted to initially examine the effect of loperamide on disease progression. Animals were treated for a maximum of 17 days based on animal ethics clearance, which is standard in order to assess both disease severity and bone damage. All manufactured formulations were applied topically on both hind paws once daily at 5 p.m., whereas Voltaren® gel was applied three times a day in accordance with the manufacturer's instructions. Fifty microliters of each formulation was applied to each paw, which is equivalent to 0.4 mg loperamide (low dose). The loperamide dose administered was based on the results from our previous study in the CFA rodent model of acute inflammatory pain (Iwaszkiewicz and Hua, 2014). In order to prevent the animals from licking the gel off soon after application, each animal was handled for ~5 min post-application by the investigator without allowing the paws to be in contact with any surfaces. This allowed enough time for the gel to be absorbed into the skin.

Paw pressure threshold (PPT), paw volume and body weight were assessed every third day (0, 3, 6, 9, 12, 15, and 17) in the morning. Testing was limited to every third day to avoid trauma to the hind paws, especially following disease onset. The time of preclinical testing and formulation application was separated (morning and afternoon, respectively) to avoid confounding the results. The only exception was for diclofenac, which needed to be applied three times a day to be effective. In this case, preclinical testing was conducted prior to application of the morning dose. The order of contralateral and ipsilateral paw testing was alternated to prevent order effects for PPT, and triplicate measurements were averaged. Inflammation was assessed with a rat plethysmometer (Ugo Basile, Comerio, Italy). This involves the placement of each paw into the displacement cell and the instrument measures displacement and interprets this as volume. Nociceptive thresholds were assessed using the paw pressure analgesiometer (Ugo Basile, Comerio, Italy), which involves a sliding weight scale and a blunt probe that places pressure on the paw against a plate surface. Animals respond by flinching or moving the paw. Cut-offs were set at 250 g for pressure threshold, which corresponds to the maximum ethical load. On the days of the preclinical testing, PPTs were measured first followed by paw volume testing. This particular order was chosen to avoid the paw volume testing procedure (involving immersion of the animal paw into a measuring tube filled with water) affecting the PPT results, whereas it is unlikely the PPT testing procedure would affect the paw volume results. Arthritis progression was scored based on the established AIA scoring protocol (Whiteley and Dalrymple, 2001). In brief, the clinical signs of inflammation were scored to evaluate the intensity of the oedema in the paws, with a score of 0 to 4 assigned to each paw for a maximum score of 16. Behavioral scoring was used as a global measure of arthritis and pain. The indicators assessed for severity (0 normal, 1 mild, 2 moderate, and 3 severe) on the adverse behavioral score sheet were vocalization on touch, weight, exploring behavior, paw ulceration, and lameness. At the end of the study, rats were sacrificed by asphyxiation with 100% CO_2_; hind paws were collected and processed for histological and immunohistological analysis.

### Histological analysis

Tissue samples were decalcified and processed for histology studies. Paraffin sections were stained with haematoxylin and eosin (H&E) (Thermo Fisher Scientific, Scoresby, Australia) to confirm the histological structure of the arthritic hind paws and to study the various features for morphology. Sections were also evaluated for cartilage proteoglycan depletion with Toluidine Blue (Sigma-Aldrich, Sydney, Australia). Histological sections were scored from 0 to 3 (0 none, 1 mild, 2 moderate, and 3 severe) by a “blinded” observer for five parameters: (i) synovitis, (ii) joint space exudate, (iii) soft tissue inflammation, (iv) cartilage degradation, and (v) bone damage. The maximum obtainable score was 15.

### Immunohistochemistry

Sections were immunostained for intercellular adhesion molecule-1 (purified mouse anti-rat ICAM-1 monoclonal antibody) (reference number 554967, BD Biosciences, Sydney, Australia) and vascular endothelial growth factor (purified mouse anti-rat VEGF monoclonal antibody) (reference number MA1-16629, Thermo Fisher Scientific, Scoresby, Australia) using standard immunohistochemistry techniques. Antigen unmasking was performed prior to antibody staining by bringing the slides to a boil in 10 mM sodium citrate buffer (pH 6.0) for 10 min. Non-specific sites were blocked by incubating slides with 5% bovine serum albumin (BSA) and 5% fetal bovine serum (FBS) in Tris Buffered Saline with Tween 20 (TBST 1X) overnight. Both primary antibodies were diluted to a working concentration of 5 μg/ml in 2% BSA and 2% FBS in TBST (1X). Slides were incubated in primary antibody overnight at 4°C in a humidity-controlled chamber. Sections were then washed in TBST (1X) buffer three times for 5 min each, prior to incubation with SignalStain® Boost IHC Detection Reagent (HRP, Mouse) (Cell Signaling Technology, Massachusetts, USA) in a humidified chamber for 1 h at room temperature. Sections were washed again in TBST (1X) wash buffer and then incubated with SignalStain® DAB (3,3′-diaminobenzidine substrate solution) (Cell Signaling Technology, Massachusetts, USA). Finally, slides were counterstained with hematoxylin (Gill's No. 2) (Sigma-Aldrich, Sydney, Australia), dehydrated and cleared in Xylene before mounting in Ultramount #4 mounting media (Thermo Scientific). Negative controls with no primary antibodies or control isotype antibodies at a concentration of 5 μg/ml (Purified Mouse IgG1, Kappa Isotype Control) (reference number 557273, BD Biosciences, Sydney, Australia) were performed on positive control slides. Sections were viewed with an Aperio® Digital Pathology Scanner (Aperio® CS2) (Leica Microsystems Pty Ltd, North Ryde, Australia). For quantification of VEGF and ICAM-1 staining, the Image J color deconvolution module was used. Thresholds were adjusted based on the no treatment control slides, with 0 to 170 used for all image analysis. The number of pixels associated with the DAB staining was then calculated (DAB area multiplied by mean pixel intensity) and then divided by the total number of pixels on the slide (Liu et al., 2016).

### Statistical analysis

All data are expressed as mean ± standard error of the mean (SEM) or standard deviation (SD). GraphPad Prism 7.01 software was used for statistical analysis. The data have been checked for normality of distribution using the D'Agostino-Pearson omnibus normality test. Comparisons between the different treatment groups over various time points were made using two-way ANOVA with Tukey's multiple comparison test (two independent variables). One-way ANOVA was used to evaluate differences between treatment groups (one independent variable). Differences were considered significant when *P* < 0.05.

## Results

### Chronic adjuvant-induced polyarthritis (AIA) model

**Figure 1:**
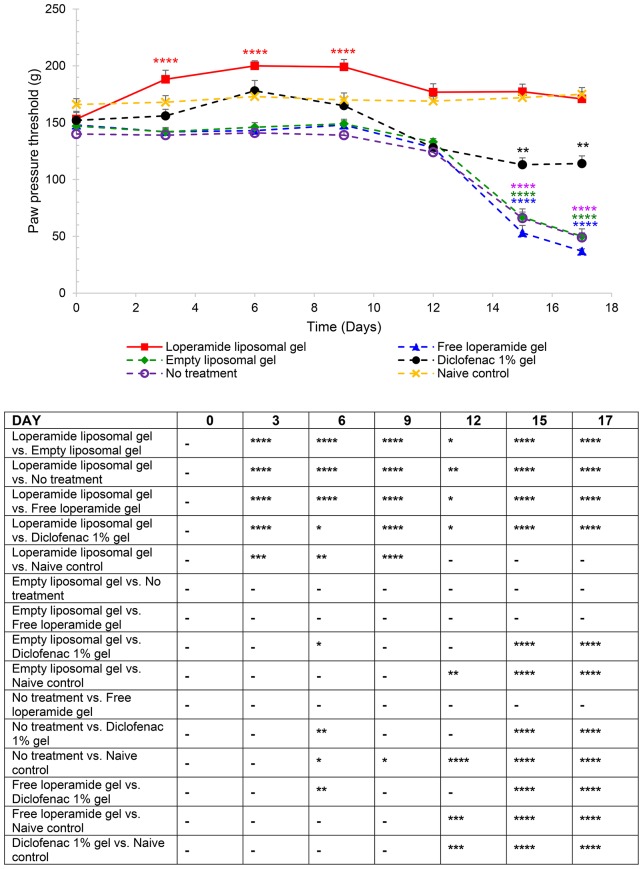
Paw pressure thresholds (PPT) of the right hind paw. The results are represented as mean ± standard error of the mean of eleven animals. Two-way ANOVA with Tukey's multiple comparison test was used to assess differences relative to baseline (refer to graph) and intergroup differences (refer to table) (^*^P < 0.05, ^**^P < 0.01, ^***^P < 0.001, ^****^P < 0.0001).

**Figure 2:**
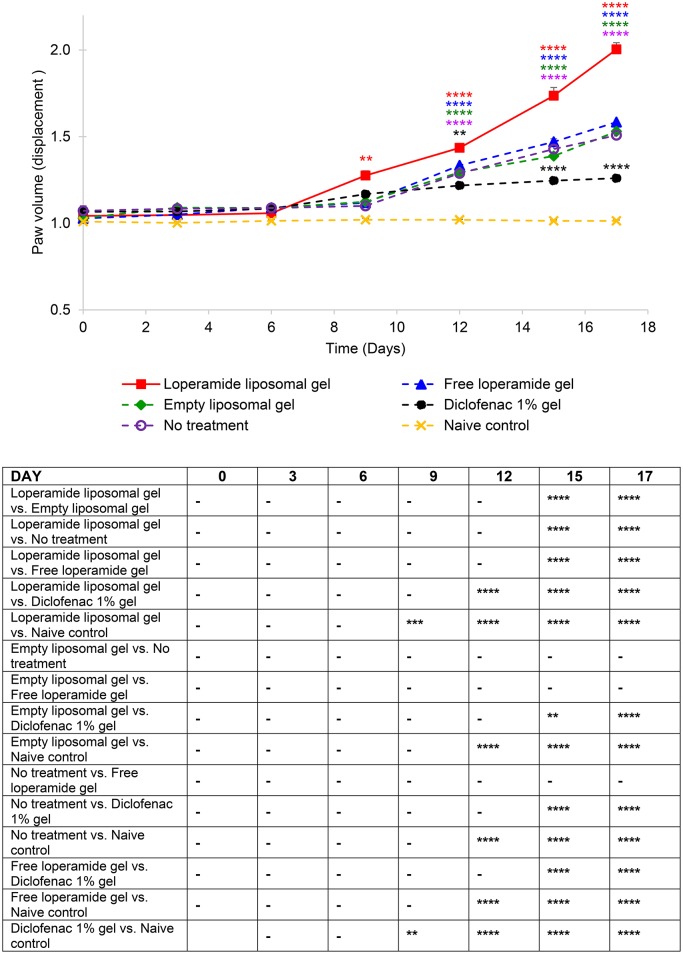
Paw volume (ml) of the right hind paw. The results are represented as mean ± standard error of the mean of eleven animals. Two-way ANOVA with Tukey's multiple comparison test was used to assess differences relative to baseline (refer to graph) and intergroup differences (refer to table) (^**^P < 0.01, ^***^P < 0.001, ^****^P < 0.0001).

**Figure 3:**
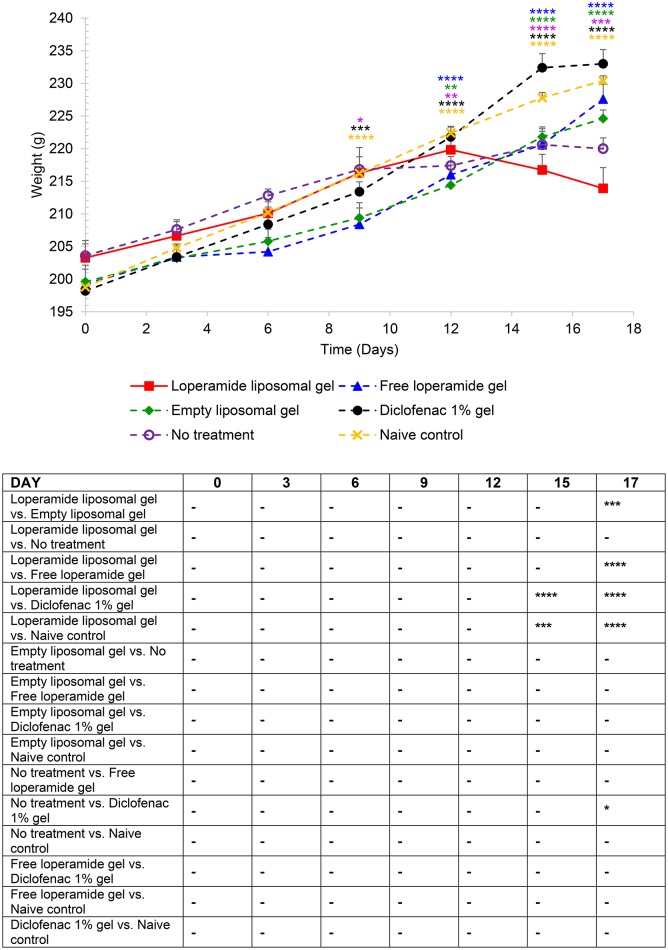
Body weight over the duration of the study. The results are represented as mean ± standard error of the mean of eleven animals. Two-way ANOVA with Tukey's multiple comparison test was used to assess differences relative to baseline (refer to graph) and intergroup differences (refer to table) (^*^P < 0.05, ^**^P < 0.01, ^***^P < 0.001, ^****^P < 0.0001).

**Figure 4:**
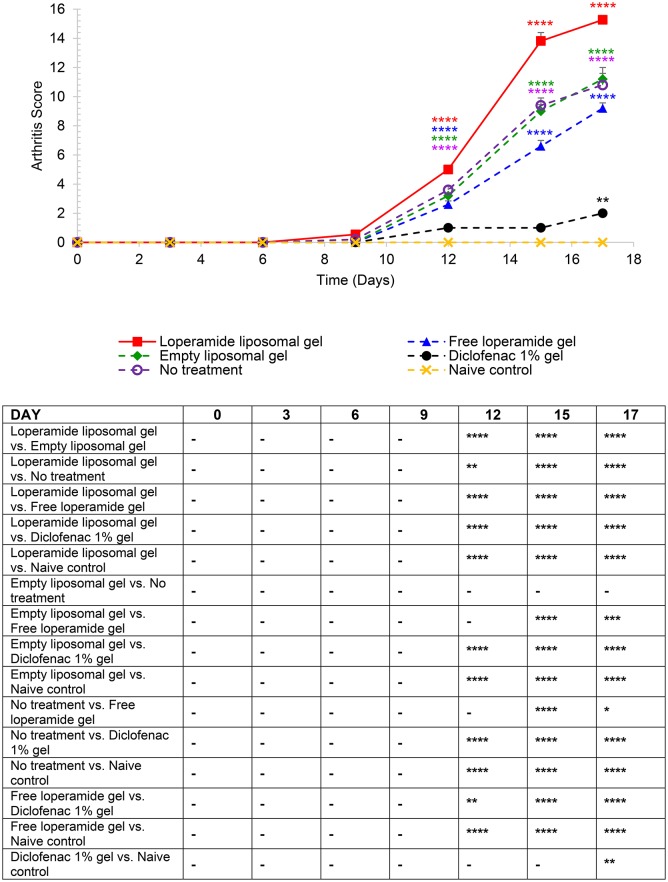
Arthritis scores based on the established AIA scoring protocol (Whiteley and Dalrymple, 2001). Each paw was scored 1 = mild, 2 = moderate, 3 = severe, 4 = very severe (total out of 16). The results are represented as mean ± standard error of the mean of eleven animals. Two-way ANOVA with Tukey's multiple comparison test was used to assess differences relative to baseline (refer to graph) and intergroup differences (refer to table) (^*^P < 0.05, ^**^P < 0.01, ^***^P < 0.001, ^****^P < 0.0001).

**Figure 5:**
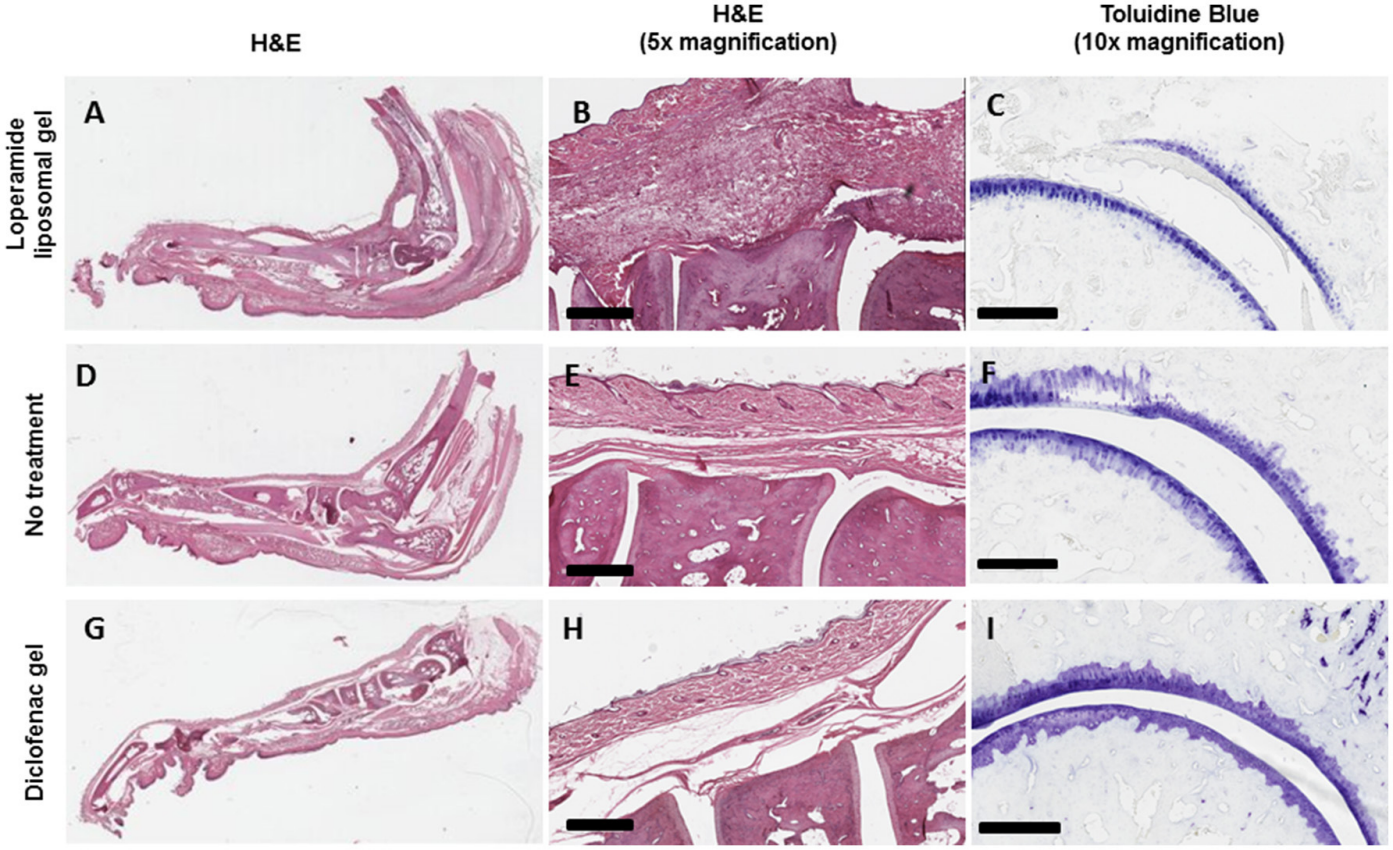
Increase in the degree of histopathologic abnormality in the joints of AIA animals treated with loperamide liposomal gel **(A–C)** compared to AIA animals administered no treatment **(D–F)** and diclofenac gel **(G–I)**. **(A,B)** Increase in pannus formulation, synovial inflammation, immune cell infiltration, and bone damage (haematoxylin and eosin, H&E). **(C)** Increase in cartilage degradation (toluidine blue). Representative pictures are shown. Scale = 500 μm (H&E) and 300 μm (toluidine blue).

**Figure 6:**
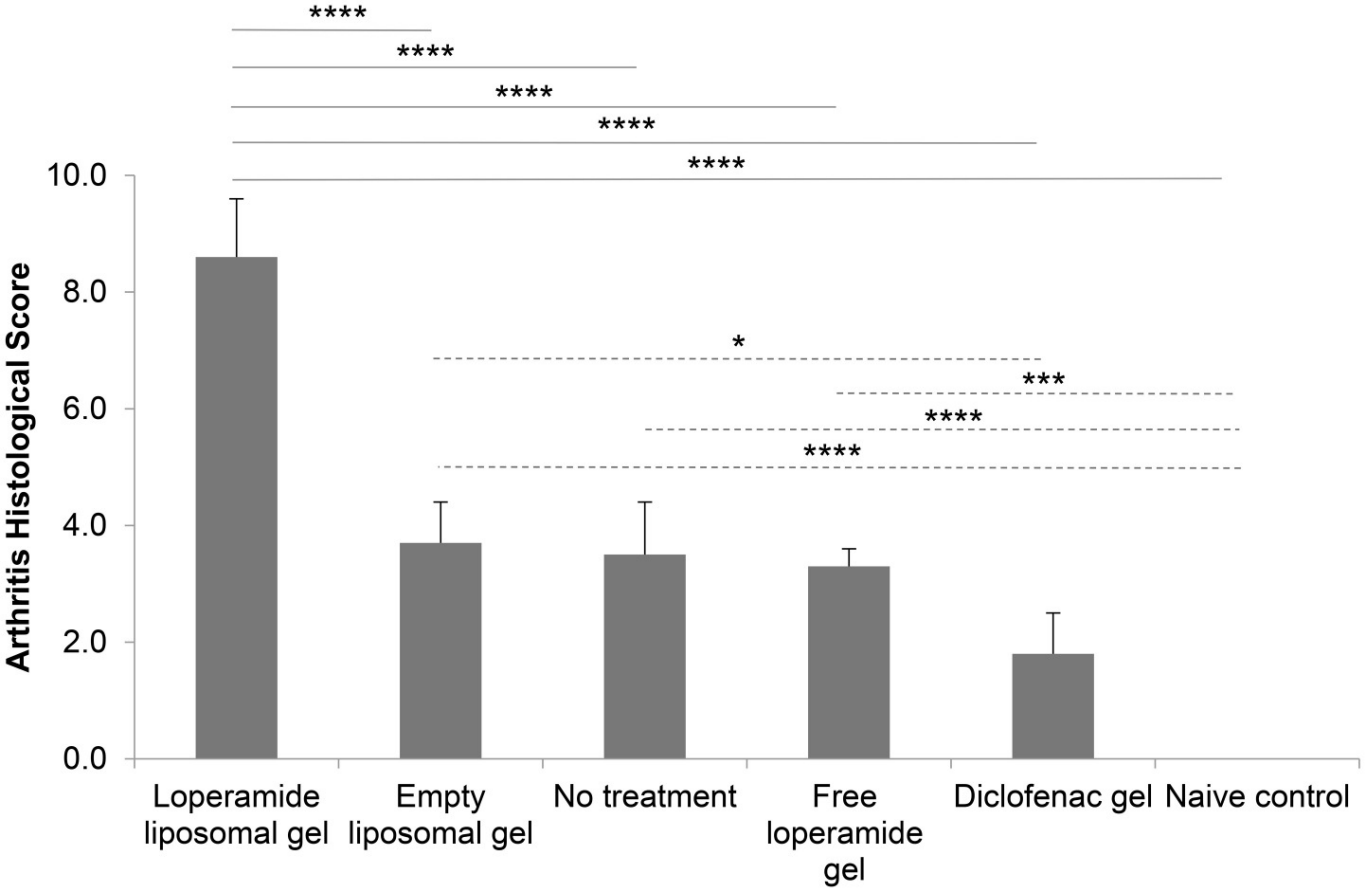
Arthritis histological scores at the end of the study. Histological sections scored from 0 to 3 for each of the following parameters—synovitis, joint space exudate, soft tissue inflammation, cartilage degradation, and bone damage (total out of 15). Sections were scored by a “blinded” observer. The results are represented as mean ± standard error of the mean of eleven animals. One-way ANOVA with Tukey's multiple comparison test was used to assess intergroup differences (^*^P < 0.05, ^***^P < 0.001, ^****^P < 0.0001).

From day 0 to 9 following the administration of 1 mg heat-killed *M. tuberculosis* at the base of the tail, the results showed that the experimental arthritic signs and symptoms (pain and inflammation) were not yet evident in the study groups compared to baseline values (Figure 1 and Figure S1, *P* > 0.05). Bilateral hyperalgesia and oedema in both hind paws started after day 9 post-inoculation (disease onset). The AIA control group receiving no treatment displayed progressive development of characteristics indicative of arthritis over this period compared to baseline values (left and right hind paw)—mean PPT of 139 ± 2.3 g (Figure 1 and Figure S1), mean paw volume of 1.07 ± 0.007 ml (Figure 2 and Figure S2), mean body weight 203 ± 2.32 g (Figure 3), and mean arthritic score of 0 (Figure 4). In particular, the mean values at day 17 of the study were PPT of 51.5 ± 2.8 g (*P* < 0.0001), paw volume of 1.53 ± 0.04 ml (*P* < 0.0001), body weight 220 ± 1.64 g (*P* < 0.001), and arthritic score of 10.80 ± 0.80 (*P* < 0.0001). Day 10 to 17 encompasses the acute clinical phase, which is consistent with human rheumatoid arthritis. This is demonstrated by progressive body weight loss (Figure 3), inflammation (Figures 2, 5, 6 and Figure S2), and cartilage degradation in the hind paws (Figures 5, 6). In comparison, the naive control group did not demonstrate any significant changes to PPT or paw volume from baseline values throughout the study (*P* > 0.05). Furthermore, the arthritis scores and histological scores do not indicate any signs of arthritis (*P* > 0.05).

### Loperamide liposomal gel induces peripheral antinociceptive effects

PPT was used as a measure of the antinociceptive effect of loperamide liposomal gel over a 17-day period, starting from day 0 at the same time as CFA immunization. Figure 1 and Figure S1 demonstrate that application of loperamide liposomal gel to both hind paws of AIA rats resulted in significant antinociception across the 17-day testing period, compared to the AIA control group receiving no treatment (*P* < 0.0001). In comparison to the naive control group, animals administered loperamide liposomal gel showed similar or higher PPTs following disease onset. There was no indication of nociception (i.e., no significant decrease in PPTs from baseline values) for either group across the duration of the study. Evaluation of the individual components of the loperamide liposomal gel (i.e., empty liposomal gel and free loperamide gel groups) showed similar results to the no treatment group (*P* > 0.05), with a significant reduction in PPT following disease onset compared to baseline values (*P* < 0.0001). These three control groups also demonstrated a significant decrease in PPTs following disease onset compared to the naive control group (175 ± 5.9 g, right hind paw; 173 ± 4.4 g, left hind paw at day 17), with mean PPT values of 50 ± 6.5 g (right hind paw) and 48 ± 3.7 g (left hind paw) for the empty liposomal gel group (*P* < 0.0001); 37 ± 2.0 g (right hind paw) and 40 ± 4.5 g (left hind paw) for the free loperamide gel group (*P* < 0.0001); and 49 ± 4.0 g (right hind paw) and 54 ± 4.0 g (left hind paw) for the no treatment group (*P* < 0.0001) at day 17. Diclofenac gel (Voltaren®) was used as a positive control (non-steroidal anti-inflammatory drug; NSAID), as it is clinically used as a topical analgesic and anti-inflammatory drug. Animals administered diclofenac gel demonstrated significant antinociceptive effects in comparison to those administered empty liposomal gel, free loperamide gel, and no treatment at day 15 and 17 (*P* < 0.0001). At the peak of disease (day 17), results showed lower PPT values for the diclofenac gel group (114 ± 6.8 g, right hind paw; 112 ± 5.8 g, left hind paw) in comparison to the loperamide liposomal group (171 ± 6.4 g, right hind paw; 170 ± 2.9 g, left hind paw) (*P* < 0.0001) and naive control group (175 ± 5.9 g, right hind paw; 173 ± 4.4 g, left hind paw) (*P* < 0.0001). In addition, no central opioid-mediated adverse effects were observed for all treatment groups in the current study.

### Loperamide liposomal gel increases the severity of inflammation

**Figure 7:**
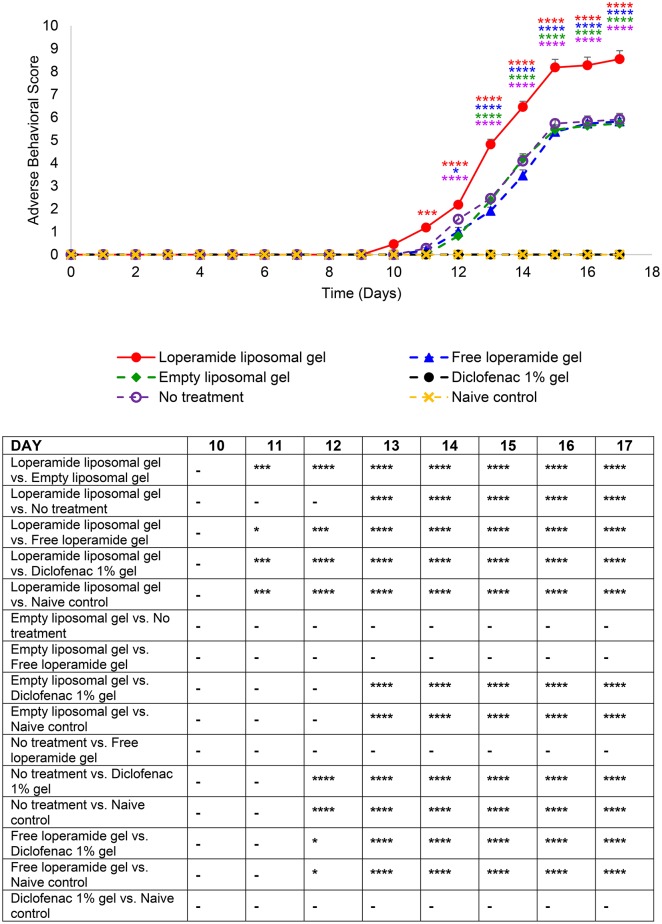
Adverse behavioral scores over the duration of the study. The indicators assessed for severity (0 normal, 1 mild, 2 moderate, and 3 severe) were vocalization on touch, weight, exploring behavior, paw ulceration, and lameness. The results are represented as mean ± standard error of the mean of eleven animals. Two-way ANOVA with Tukey's multiple comparison test was used to assess differences relative to baseline (refer to graph) and intergroup differences (refer to table) (^*^P < 0.05, ^***^P < 0.001, ^****^P < 0.0001).

Paw volume was used as an indicator of the anti-inflammatory efficacy of the loperamide liposomal formulation over the duration of the study. The loperamide liposomal gel group displayed significantly increased inflammation compared to all control groups (empty liposomal gel, no treatment, free loperamide gel, diclofenac gel, and naive control) (Figure 2 and Figure S2). In particular, the loperamide liposomal gel group had mean paw volume values of 2.00 ± 0.04 ml (right hind paw) and 1.97 ± 0.04 ml (left hind paw) at day 17, whereas the no treatment group had mean paw volume values of 1.51 ± 0.06 ml (right hind paw) and 1.54 ± 0.07 ml (left hind paw) (*P* < 0.0001). The naive control group showed no significant paw volume changes from baseline values (1.01 ± 0.01 ml, right hind paw; 1.01 ± 0.004 ml, left hind paw) throughout the study duration, with mean paw volume values of 1.01 ± 0.01 ml (right hind paw) and 1.02 ± 0.01 ml (left hind paw) at day 17. The free loperamide gel and empty liposomal vehicle had no effect on inflammation on their own, as indicated by similar mean paw volume results to the AIA animals receiving no treatment (*P* > 0.05). Animals administered diclofenac gel displayed significant anti-inflammatory activity in comparison to those administered loperamide liposomal gel, free loperamide gel, empty liposomal gel, and no treatment (*P* < 0.0001). In comparison to baseline mean paw volume values of 1.07 ± 0.03 ml (right hind paw) and 1.09 ± 0.05 ml (left hind paw), the diclofenac gel group still demonstrated signs of inflammation at the peak of disease with mean paw volume values of 1.26 ± 0.01 ml (right hind paw) and 1.23 ± 0.02 ml (left hind paw) at day 17 (*P* < 0.0001). When compared to the naive control group, animals administered diclofenac gel displayed higher mean paw volume values following disease onset (*P* < 0.0001). Figure 7 represents the mean adverse behavioral scores for each treatment group across the duration of the study. At the peak of disease, animals administered loperamide liposomal gel demonstrated significantly higher adverse behavioral scores compared to all other control groups (*P* < 0.0001). The loperamide liposomal gel group displayed limited vocalization on touch, significantly reduced exploring behavior or movement, paw ulceration, and moderate-to-severe lameness. Animals administered diclofenac gel and the naive control group showed no adverse behavioral signs indicative of pain or arthritis throughout the study (*P* > 0.05).

### Loperamide liposomal gel accelerates progression of experimental arthritis

AIA animals administered loperamide liposomal gel demonstrated accelerated disease progression in comparison to the control groups (empty liposomal gel, free loperamide gel, diclofenac gel, no treatment and naive control) as indicated by (i) reduction in body weight over the duration of the study (Figure 3), (ii) high overall arthritis scores based on the established AIA scoring protocol (Whiteley and Dalrymple, 2001) (Figure 4), and (iii) high overall arthritis histological score at the end of the study (Figure 6). Reduction in body weight was evident in the loperamide liposomal gel group at the peak of disease, with a mean body weight of 213 ± 3.18 g at day 17. This is significantly lower compared to the naive control group (230 ± 0.75 g), empty liposomal gel group (224 ± 1.33 g), free loperamide gel group (227 ± 2.29 g), and diclofenac gel group (233 ± 2.19 g). The control groups (empty liposomal gel, free loperamide gel, diclofenac gel, no treatment, and naive control) showed an increase in body weight over the duration of the study compared to baseline values (*P* < 0.001). Although the no treatment control group showed a progressively slower increase in body weight following the onset of disease, the mean body weight at day 17 (220 ± 1.64 g) was not considered significant compared the loperamide liposomal gel group (*P* > 0.05). Animals administered diclofenac gel had similar body weight values as the naive control group throughout the study, however these values were not significantly different from the other control groups (*P* > 0.05). The only exception was at day 17 between the diclofenac gel and no treatment group, where the animals administered diclofenac gel showed higher body weight values (*P* < 0.05).

Mean arthritis scores in the animals administered loperamide liposomal gel was significantly higher following disease onset compared to baseline, with a mean peak at day 17 of 15.27 ± 0.30 (Figure 4, *P* < 0.0001). The empty liposomal gel, free loperamide gel and no treatment groups showed lower arthritis scores in comparison to the loperamide liposomal gel group, with a mean peak at day 17 of 11.20 ± 0.80, 9.20 ± 0.37, and 10.80 ± 0.80, respectively (*P* < 0.0001). There was no significant difference in arthritis scores between the empty liposomal gel and no treatment group (*P* > 0.05). The free loperamide gel group showed slightly lower arthritis scores compared to the empty liposomal gel group (day 17, *P* < 0.001) and no treatment group (day 17, *P* < 0.05). Animals administered diclofenac gel displayed low arthritis scores throughout the study compared to baseline, with a maximum score of 2 at the peak of disease (*P* < 0.01). The mean arthritis score for the diclofenac gel group at day 17 was significantly higher compared to the naive control group, which showed no signs of arthritis (*P* < 0.01). In addition, the arthritis scores for the diclofenac gel group was significantly lower following disease onset in comparison to the loperamide liposomal gel, empty liposomal gel, free loperamide gel, and no treatment groups (*P* < 0.0001).

Histological analysis of the morphology of the joints showed that the animals treated with loperamide liposomal gel had increased pannus formation, synovial inflammation, cartilage degradation, and bone erosion (Figures 5A–C) compared to the no treatment group (Figures 5D–F) and diclofenac gel group (Figures 5G–I). This is further supported by the histological scores of the severity of arthritis (Figure 6), with the loperamide liposomal gel group having a mean score of 8.6 ± 1.0 compared to the control groups receiving empty liposomal gel (3.7 ± 0.7, *P* < 0.0001), no treatment (3.5 ± 0.9, *P* < 0.0001), free loperamide gel (3.3 ± 0.3, *P* < 0.0001), diclofenac gel (1.8 ± 0.7, *P* < 0.0001), and the naive control group (0, *P* < 0.0001). Animals administered empty liposomal gel, free loperamide gel, and no treatment had similar arthritis histological scores. Those administered diclofenac gel displayed significantly lower histological scores compared to the empty liposomal gel group (*P* < 0.05). There was no difference between the arthritis histological scores for the diclofenac gel group and naive control group (*P* > 0.05). In comparison to the naive control group, the empty liposomal gel group, free loperamide gel group, and no treatment group displayed higher arthritis histological scores (*P* < 0.001).

**Figure 8:**
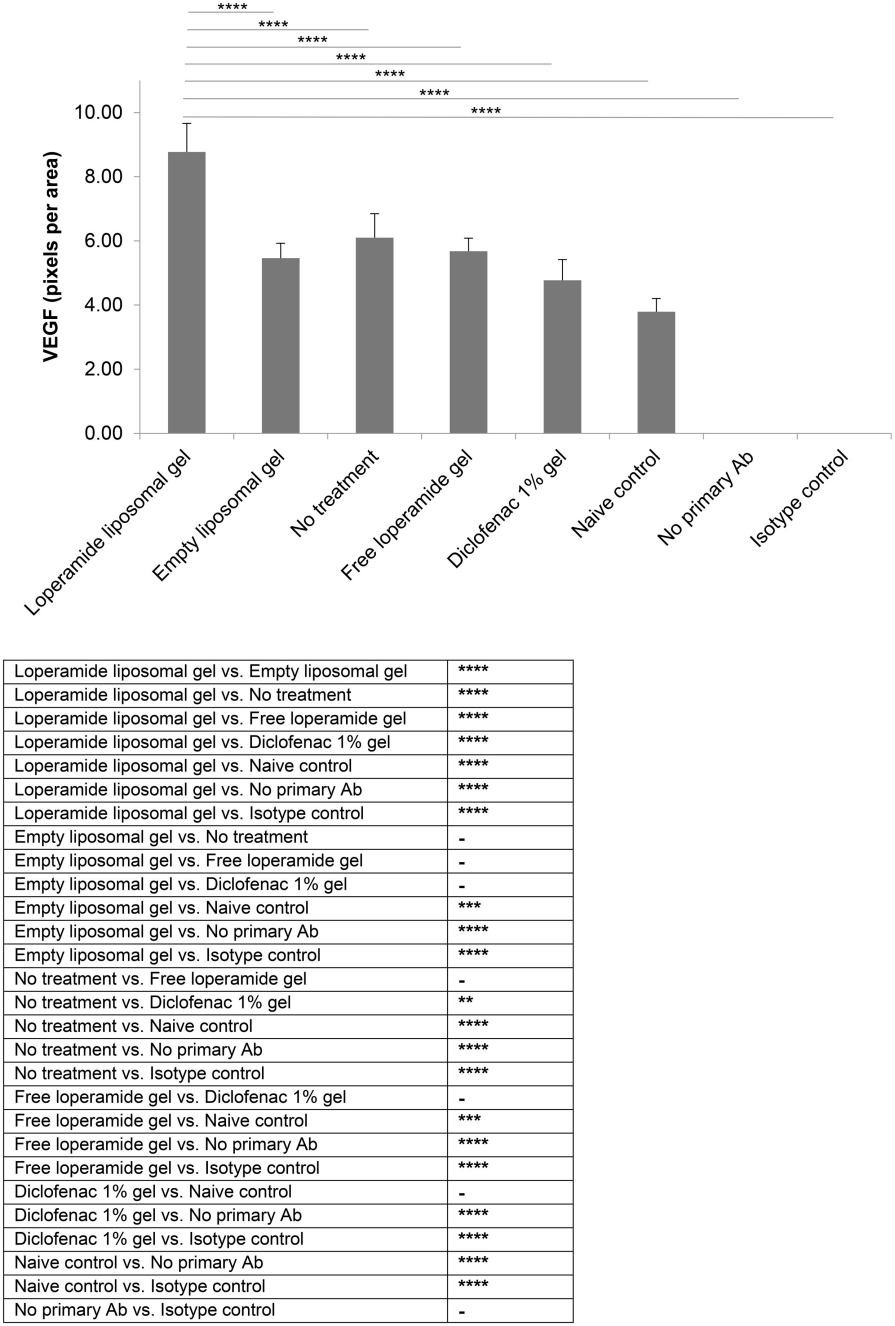
VEGF expression. Tissue samples were collected, decalcified and processed for immunohistochemistry at day 17 of the study. Staining intensity for VEGF based on the number of pixels associated with the DAB staining. The results are represented as mean ± standard deviation of eleven animals. One-way ANOVA with Tukey's multiple comparison test was used to assess intergroup differences (^**^P < 0.01, ^***^P < 0.001, ^****^P < 0.0001).

**Figure 9:**
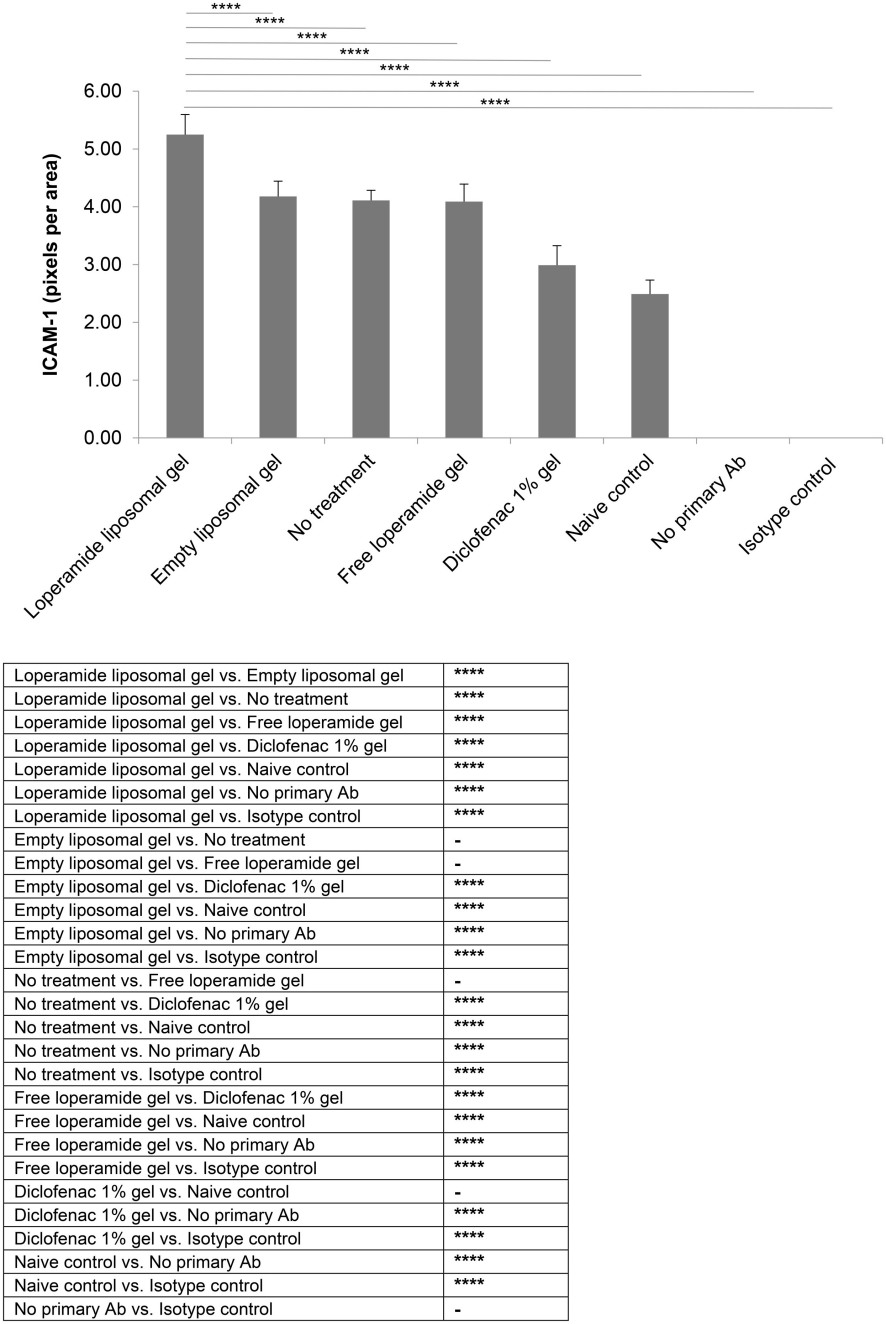
ICAM-1 expression. Tissue samples were collected, decalcified and processed for immunohistochemistry at day 17 of the study. Staining intensity for ICAM-1 based on the number of pixels associated with the DAB staining. The results are represented as mean ± standard deviation of eleven animals. One-way ANOVA with Tukey's multiple comparison test was used to assess intergroup differences (^****^P < 0.0001).

Immunohistological analysis for VEGF (Figure 8) and ICAM-1 (Figure 9), two markers of inflammation and disease severity, showed increased expression in the loperamide liposomal gel group in comparison to the control groups (empty liposomal gel, free loperamide gel, no treatment, diclofenac gel, and naive control) (*P* < 0.0001). Animals administered empty liposomal gel, free loperamide gel, and no treatment had similar VEGF and ICAM-1 expressions (*P* > 0.05). In addition, those administered diclofenac gel showed a significant decrease in ICAM-1 expression compared to the control groups (empty liposomal gel, free loperamide gel, and no treatment) (*P* < 0.0001). For VEGF, diclofenac gel showed a significant decrease in expression when compared to the no treatment control group (*P* < 0.01). No difference was seen between the diclofenac gel and naive control groups for either VEGF or ICAM-1 expression (*P* > 0.05). Furthermore, no labeling was observed with the negative controls where the primary antibody was omitted or when using control isotype antibodies (Purified Mouse IgG1, Kappa Isotype Control).

## Discussion

The effects of opioids outside of the CNS are only beginning to be elucidated, following the identification of opioid receptors on peripheral tissues and cells (Philippe et al., 2003; Chakass et al., 2007; Smith et al., 2007; Hua and Cabot, 2010; Stein and Kuchler, 2012; Stein and Küchler, 2013). Peripheral opioid receptor-mediated analgesia has been widely demonstrated in patients (Stein et al., 1991, 1993, 1999, 2003; Nozaki-Taguchi and Yaksh, 1999; Rittner et al., 2005; Nozaki-Taguchi et al., 2008; van Ingen et al., 2008; Labuz et al., 2009; Hua and Cabot, 2010; Iwaszkiewicz et al., 2013); however, the effect of peripheral opioids on inflammation has only recently been studied (Philippe et al., 2003; Chakass et al., 2007; Smith et al., 2007; Stein and Kuchler, 2012; Hua and Cabot, 2013; Iwaszkiewicz and Hua, 2014). To determine the place for peripheral opioid analgesics in the clinic, it is important to understand the overall effects of these agents in acute vs. chronic inflammatory pain. Although morphine has also been shown to have peripheral analgesic effects, such opioids are associated with considerable adverse effects and high abuse potential, owing to their central opioid-mediated activity. Therefore, our recent studies have been focused on the use of loperamide HCl, which is currently the only peripherally-selective MOR agonist on the market and has a long history of safety, due to its poor bioavailability and minimal CNS penetration. Its propensity for abuse is significantly lower compared to conventional opioids, however extremely high doses have been reported to lead to cardiac dysrhythmia (Eggleston et al., 2017).

Loperamide does not have analgesic effects when administered topically on intact skin, orally, or intravenously due to its physicochemical properties. Loperamide displays high affinity to lipid membranes, an ability to decrease surface tension, and is actively removed by the multi-drug resistance transporter (which minimizes its distribution into the CNS) (Heel et al., 1978; DeHaven-Hudkins et al., 1999; Stein et al., 2001; Sevostianova et al., 2005). This contributes to its accumulation in membranes and subsequent lack of systemic absorption (Heel et al., 1978; Stein et al., 2001). Following topical application to intact skin, loperamide associates within the stratum corneum and cannot penetrate further due to its lipophilic nature. This restricts it from penetrating into deeper layers where peripheral opioid receptors are expressed (Regnard et al., 2011). Hence loperamide in the free drug form does not have any effect on pain or inflammation when applied topically on intact skin (Iwaszkiewicz and Hua, 2014). The addition of penetration enhancers (e.g., propylene glycol) in the topical formulation base still does not readily improve the dermal delivery of loperamide, with *in vitro* studies over-estimating its efficacy *in vivo* (Trottet et al., 2004). Therefore, drug delivery formulation is required to investigate the use of loperamide as a topical analgesic.

We have previously used liposomes as a delivery mechanism to enhance the topical permeability of loperamide across painful conditions involving intact skin (Iwaszkiewicz and Hua, 2014), and intravenous delivery of loperamide to peripheral sites of inflammation (Hua and Cabot, 2013). It should be noted that the liposome vehicle itself is inert, thus having no effect on pain or inflammation. Using the CFA model of acute inflammatory pain (unilateral hind paw inflammation), which is similar to an acute soft tissue injury, administration of liposomal formulations of loperamide resulted in potent antinociceptive and anti-inflammatory activity in peripheral tissues (Hua and Cabot, 2013; Iwaszkiewicz and Hua, 2014). These results are in agreement with published data (Stein and Kuchler, 2012). In particular, a single intravenous dose of loperamide-encapsulated ICAM-1 targeted immunoliposomes (0.8 mg) was able to produce significant and prolonged antinociceptive and anti-inflammatory actions over a 48 h study duration in comparison to control groups (loperamide-encapsulated non-targeted liposomes, empty anti-ICAM-1 immunoliposomes, empty non-targeted liposomes, and loperamide solution) (Hua and Cabot, 2013). Similar results were also attained using topical application of loperamide liposomal gel in the CFA model compared to control groups (empty liposomal gel, diclofenac gel, and free loperamide gel) (Iwaszkiewicz and Hua, 2014). Antinociception was able to be reversed in both studies via intraplantar injection of naloxone methiodide (1 mg/kg; peripheral MOR antagonist) 15 min prior to administration of the loperamide formulation, therefore suggesting a MOR-dependent antinociceptive effect (Hua and Cabot, 2013; Iwaszkiewicz and Hua, 2014). As naloxone methiodide has a short duration of action (~4 h) it was not expected to affect the anti-inflammatory response. In addition, the loperamide liposomal formulations did not affect the PPTs or paw volumes of the non-inflamed hind paws following both intravenous systemic administration or local topical application across intact skin in the CFA model (Hua and Cabot, 2013; Iwaszkiewicz and Hua, 2014).

Based on the positive results of our previous studies using liposomal loperamide in the treatment of acute peripheral inflammatory pain, we expected to see similar results in the AIA model of experimental RA (chronic peripheral inflammatory pain). The AIA model is an immune-mediated joint inflammation (polyarthritis) whose histopathology shows many similarities to human RA and is a widely used model in preclinical testing of new agents for RA (Whiteley and Dalrymple, 2001; Bolon et al., 2011). Peripheral opioid receptors are upregulated on synovial cells, chondrocytes, peripheral nerve fibers and activated immune cells in RA, thus making it an appropriate target (Mousa et al., 2007). We used the topical liposomal formulation for this study, as it is more applicable in a clinical setting to manage chronic pain compared to intravenous dosing. Prophylactic administration of loperamide liposomal gel daily across the duration of the study in the AIA model produced significant peripheral analgesia as expected. Once daily application of loperamide liposomal gel demonstrated to be a more effective antinociceptive agent compared to standard topical NSAIDs that require application three times a day. However, loperamide liposomal gel increased the severity of inflammation and accelerated arthritis progression. This was indicated by an increase in paw volume, behavioral and observational scoring, and histological analysis compared to the no treatment AIA control group. In particular, histology results showed an increase in pannus formation, synovial inflammation, cartilage degradation and bone erosion, as well as an upregulation of ICAM-1 and VEGF—markers of inflammation and angiogenesis, respectively. These characteristics correlate with inflammation and disease severity (Taylor, 2002; Nigrovic and Lee, 2005; Ng et al., 2010). As expected from our previous studies (Hua and Cabot, 2013; Iwaszkiewicz and Hua, 2014), the individual components of the loperamide liposomal gel (free loperamide gel and empty liposomal gel) showed similar results to the no treatment AIA control group. In addition, animals administered diclofenac gel demonstrated significant anti-inflammatory activity compared to the loperamide liposomal gel, empty liposomal gel, free loperamide gel, and no treatment groups. However, signs of pain and inflammation were still evident for the diclofenac gel group when compared to the naive control group throughout the study.

The interplay of opioids in arthritis, angiogenesis and inflammation is not yet fully understood. Inflammation and angiogenesis are closely integrated processes in arthritis, and may affect disease progression and pain. Inflammation can stimulate angiogenesis, and angiogenesis can facilitate inflammation (Ng et al., 2010; Konisti et al., 2012; Hua and Dias, 2016). Synovial inflammation exacerbates structural damage in RA and leads to a poor clinical outcome (Ng et al., 2010; Konisti et al., 2012; Hua and Dias, 2016). The mechanisms by which synovitis exacerbates disease progression in arthritis are likely to be complex. This study shows that analgesia is maintained following peripheral opioid use in experimental RA; however, there is an overall shift toward a detrimental response in peripheral tissues. Two preclinical animal studies have shown that the peripheral effects of opioids in RA is anti-inflammatory—one study was focused on kappa opioid receptors (Binder and Walker, 1998), and the other study administered endomorphin (MOR agonist) short-term and lacked adequate control groups (Straub et al., 2008). In particular, Straub et al. administered a single intraperitoneal injection of endomorphin-1 (0.1 μmol or 1 μmol) or saline control on days 9 to 13 in AIA polyarthritis animals (*n* = 6) (Straub et al., 2008). The study did not assess antinociceptive effects and only measured paw volume as the indicator of anti-inflammatory activity. In addition, no opioid antagonist was evaluated in the study. The reason for the discrepancy in our results to those of the other studies is unknown and may be related to the specificity for MOR, duration of administration, or additional mechanisms of action of the compound.

Contradicting results have been reported in the literature, with studies having demonstrated anti-inflammatory activity of opioid receptor agonists (Binder and Walker, 1998; Philippe et al., 2003; Hua and Cabot, 2013; Stein and Küchler, 2013; Iwaszkiewicz and Hua, 2014), proinflammatory activity of opioid receptor agonists (Peng et al., 2000; Vujic et al., 2004), and anti-inflammatory activity of opioid receptor antagonists (Greeneltch et al., 2004; Smith et al., 2007) in peripheral tissues. Several mechanisms of action for the anti-inflammatory effects of opioid agonists have been suggested, including preventing the vesicular release of noradrenaline and substance P from neuronal cells (O'Connor et al., 2004; Heneka et al., 2010; Schlachetzki et al., 2010), inhibiting tumor necrosis factor (TNF) production and release (Walker, 2003), and reducing neuroimmune adhesion between immune cells and peripheral sensory neurons (Hua et al., 2006). An opioid receptor independent mechanism may also be involved in opioid-mediated anti-inflammation. Gavalas et al. showed that experimentally induced mouse paw oedema was significantly inhibited after the administration of opioids and this effect was not reversed by naxolone (Gavalas et al., 1994). In addition, Fecho et al. demonstrated an anti-inflammatory action of morphine through the reduction of swelling and accumulation of neutrophils in carrageenan-induced peripheral inflammation (Fecho et al., 2007). This effect was not dose-dependent and was not reversed by naloxone (Fecho et al., 2007). The anti-inflammatory effect displayed by morphine is likely due to modulation of the adherence of immune cells to the endothelium by affecting the expression of cell adhesion molecules, and consequently affecting leukocyte transmigration (Fecho et al., 2007). Conversely, Philippe et al. showed that naloxone was able to reverse the MOR-mediated reduction in inflammation in two *in vivo* models of colitis (Philippe et al., 2003).

Several studies have reported pro-inflammatory effects of opioid receptor agonists and anti-inflammatory effects of opioid receptor antagonists. Peng et al. showed that morphine enhanced interleukin-12 and the production of other pro-inflammatory cytokines in mouse peritoneal macrophages, which was reversed by naltrexone (Peng et al., 2000). It was suggested that the enhancement of IL-12 by morphine might be related to morphine-induced sepsis (Peng et al., 2000). Similarly, methionine-enkephalin has been shown to modulate various functions of macrophages related to both immune and inflammatory reactions in an opioid receptor dependent manner, including stimulating hydrogen peroxide and nitric oxide production in rat peritoneal macrophages (Vujic et al., 2004; Stanojevic et al., 2008). This suggests that opioid receptors are involved in the regulation of macrophage activity (Vujic et al., 2004; Stanojevic et al., 2008). Correspondingly, opioid receptor antagonism with naltrexone has been shown to block TNF-α production in a murine model of acute endotoxic shock (Greeneltch et al., 2004), as well as have direct mucosal healing activity (Zagon et al., 1997, 2002; Zagon and McLaughlin, 2005). In particular, administration of naltrexone protected mice from shock induced by lipopolysaccharide (LPS) with D-galactosamine (D-gal) to significantly inhibit the production of TNF-α, and this was reversed with morphine (Greeneltch et al., 2004). Interestingly, when bone marrow-derived, splenic or peritoneal macrophages were treated with LPS *in vitro*, administration of naltrexone had no direct effect on TNF-α production (Greeneltch et al., 2004). Therefore, naltrexone may prevent LPS-induced septic shock mortality by indirect inhibition of TNF-α production *in vivo*. Overall, the results to date demonstrate that a variety of complex regulatory activities may be performed by opioid agonists and antagonists in various tissues of the body, and these pathways may directly or indirectly modulate the release of cytokines and mediators involved in inflammation.

Although the analgesic effects of loperamide liposomal gel are likely acting through MORs based on our previous studies (Hua and Cabot, 2013; Iwaszkiewicz and Hua, 2014), we have yet to determine whether the detrimental effects are occurring through the same pathway to increase arthritis severity and accelerate disease progression. Investigating the pathways that cause the exacerbation of RA with peripheral opioid use in preclinical and clinical studies is the goal of our ongoing studies. In addition, comparing prophylaxis vs. treatment with loperamide and other conventional opioids is also necessary. In order to conduct these studies, several challenges will firstly need to be addressed, including specificity for MORs and drug administration issues for chronic administration of the conventional opioid agonists and antagonists for peripheral opioid receptor targeting. In particular, systemic opioid receptor antagonists (e.g., naloxone hydrochloride and naltrexone) cannot be used to ascertain peripheral opioid mechanisms, as they will block both central and peripheral MORs. In addition, naloxone methiodide is a peripherally selective MOR antagonist, however has a short duration of action and requires administration via intraplantar injection. The need for multiple local injections daily will damage the paw tissues and confound the results. We have previously considered using implantable minipumps, however the subcutaneous implantation of the pump and catheter to deliver the compounds specifically to the tissues of both hind paws was considered invasive, and thus likely to also confound the results of the study that is focused on pain and inflammation. Therefore, we are working on developing a topical peripheral opioid receptor antagonist formulation that can be applied chronically to intact skin of AIA animals and allow prolonged drug release, in order to evaluate the proinflammatory mechanism of loperamide. A topical dosage form is optimal for direct comparison in this animal model and across the duration of the study. It should be noted that the use of highly selective antagonists to the opioid receptor subtypes will be restricted to *in vitro* studies due to the high costs of the drugs at the *in vivo* doses required. Although this initial study is focused on the prophylactic use of loperamide in experimental RA, the findings may have implications for the use of loperamide and other opioids in RA and potentially other chronic inflammatory diseases.

## Author contributions

All authors were involved in revising the article critically for important intellectual content. Drafting of manuscript: SH. Study conception and design: SH. Acquisition of data: SH, TD, and DP. Analysis and interpretation of data: SH, DP, and YY.

### Conflict of interest statement

The authors declare that the research was conducted in the absence of any commercial or financial relationships that could be construed as a potential conflict of interest.

## Supplementary material

Figure S1 Paw pressure thresholds (PPT) of the left hind paw. The results are represented as mean ± standard error of the mean of eleven animals. Two-way ANOVA with Tukey's multiple comparison test was used to assess differences relative to baseline (refer to graph) and intergroup differences (refer to table) (^*^*P* < 0.05, ^**^*P* < 0.01, ^****^*P* < 0.0001).

Figure S2 Paw volume (ml) of the left hind paw. The results are represented as mean ± standard error of the mean of eleven animals. Two-way ANOVA with Tukey's multiple comparison test was used to assess differences relative to baseline (refer to graph) and intergroup differences (refer to table) (^*^*P* < 0.05, ^**^*P* < 0.01, ^***^*P* < 0.001, ^****^*P* < 0.0001).
