# Supplementary material for: Topical Loperamide-Encapsulated Liposomal Gel Increases the Severity of Inflammation and Accelerates Disease Progression in the Adjuvant-Induced Model of Experimental Rheumatoid Arthritis
Source: Front Pharmacol. 2017 Aug 2;8:503. doi: 10.3389/fphar.2017.00503 (PMC5539122; doi:10.3389/fphar.2017.00503)
Supplement: Figure S2 — Paw volume (ml) of the left hind paw. The results are represented as mean ± standard error of the mean of eleven animals. Two-way ANOVA with Tukey's multiple comparison test was used to assess differences relative to baseline (refer to graph) and intergroup differences (refer to table) (*P < 0.05, **P < 0.01, ***P < 0.001, ****P < 0.0001). [file Image2.pdf]

Figure S2

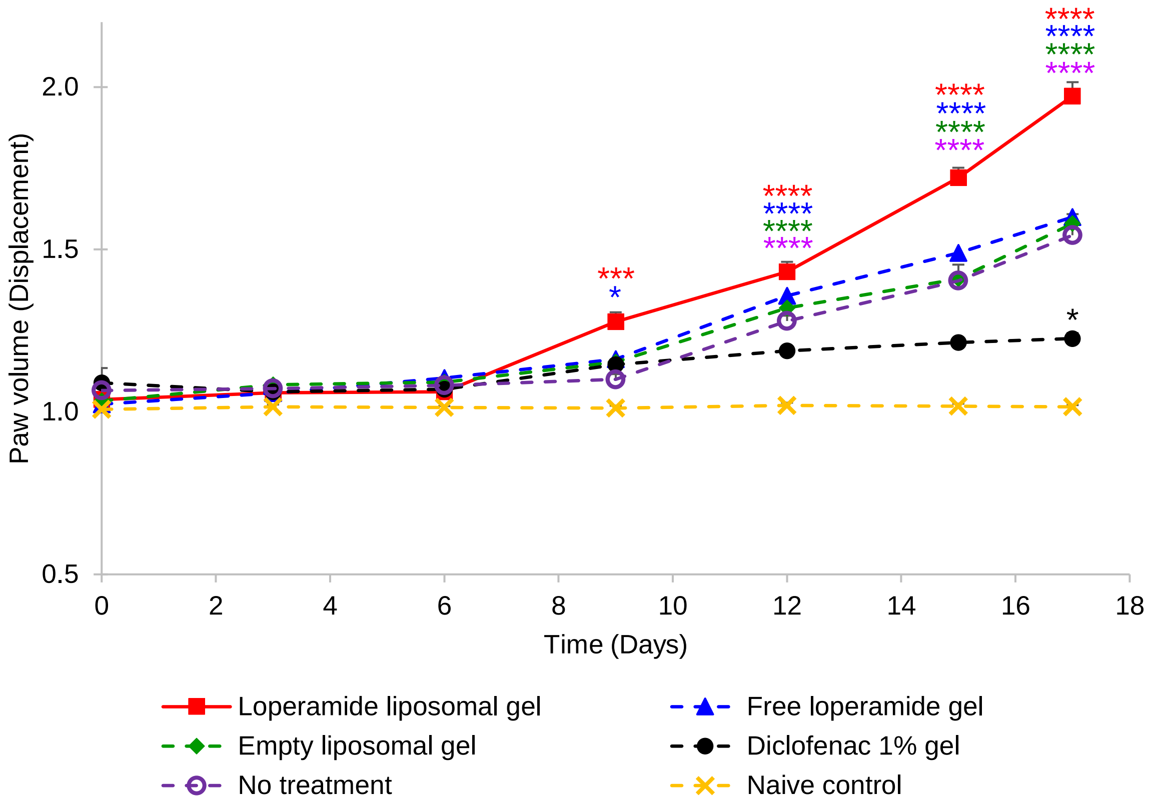

DAY	0	3	6	9	12	15	17
Loperamide liposomal gel vs. Empty liposomal gel	-	-	-	-	-	****	****
Loperamide liposomal gel vs. No treatment	-	-	-	-	-	****	****
Loperamide liposomal gel vs. Free loperamide gel	-	-	-	-	-	****	****
Loperamide liposomal gel vs. Diclofenac 1% gel	-	-	-	-	***	****	****
Loperamide liposomal gel vs. Naive control	-	-	-	***	****	****	****
Empty liposomal gel vs. No treatment	-	-	-	-	-	-	-
Empty liposomal gel vs. Free loperamide gel	-	-	-	-	-	-	-
Empty liposomal gel vs. Diclofenac 1% gel	-	-	-	-	*	****	****
Empty liposomal gel vs. Naive control	-	-	-	**	****	****	****
No treatment vs. Free loperamide gel	-	-	-	-	-	-	-
No treatment vs. Diclofenac 1% gel	-	-	-	-	-	****	****
No treatment vs. Naive control	-	-	-	-	****	****	****
Free loperamide gel vs. Diclofenac 1% gel	-	-	-	-	***	****	****
Free loperamide gel vs. Naive control	-	-	-	**	****	****	****
Diclofenac 1% gel vs. Naive control	-	-	-	*	***	****	****
